# S2A: Scale-Attention-Aware Networks for Video Super-Resolution

**DOI:** 10.3390/e23111398

**Published:** 2021-10-25

**Authors:** Taian Guo, Tao Dai, Ling Liu, Zexuan Zhu, Shu-Tao Xia

**Affiliations:** 1 College of Computer Science and Software Engineering, Shenzhen University, Shenzhen 518060, China; gta17@mails.tsinghua.edu.cn (T.G.); liulingcs@szu.edu.cn (L.L.); zhuzx@szu.edu.cn (Z.Z.); 2 Tsinghua Shenzhen International Graduate School, Tsinghua University, Beijing 100084, China; xiast@sz.tsinghua.edu.cn

**Keywords:** scale-and-attention-aware, criss-cross channel attention, video super-resolution

## Abstract

Convolutional Neural Networks (CNNs) have been widely used in video super-resolution (VSR). Most existing VSR methods focus on how to utilize the information of multiple frames, while neglecting the feature correlations of the intermediate features, thus limiting the feature expression of the models. To address this problem, we propose a novel SAA network, that is, Scale-and-Attention-Aware Networks, to apply different attention to different temporal-length streams, while further exploring both spatial and channel attention on separate streams with a newly proposed Criss-Cross Channel Attention Module ($C^{3}AM$). Experiments on public VSR datasets demonstrate the superiority of our method over other state-of-the-art methods in terms of both quantitative and qualitative metrics.

## 1. Introduction

As a fundamental problem in low-level vision, Super-Resolution (SR) has drawn more and more focus in recent years. Unlike single image SR, which can only utilize the internal spatial information of a single image, video SR takes the consecutive frames sequence as input and, thus, information gain can be achieved with the aid of redundant temporal correlations extracted from the neighboring frames. In the meantime, how to effectively extract multi-frame information and utilize the correlations between multiple frames remains a challenging problem for video SR.

Based on the method of using multi-frames, recent deep learning based video SR methods can be divided into several categories. Some methods utilize the multiple input frames in a frame-recurrent way [1,2,3], taking the input frames as a temporal frames sequence, which is processed by the network one by one. Several recent works [4,5,6] attempt to exploit correlations of multi-frames to improve video super-resolution. Yi et al. [4] proposed a progressive fusion network by exploiting non-local spatial–temporal correlations. Isobe et al. [5] proposed an effective video super-resolution by incorporating temporal information in a hierarchical way. Later, Zhao et al. [6] proposed an efficient space–time distillation scheme to exploit both spatial and temporal knowledge in the video super-resolution task. Others fuse the frames in some way to obtain a fused feature for further feature extraction and reconstruction process. Specifically, direct or slow concatenation [7,8,9], 3D convolution [10], and progressive fusion [4] have been taken into consideration. While these methods provide various probabilities to utilize the multiple frames, they neglect the correlations between extracted features of multi-frames.

To address this problem, we propose a Scale-and-Attention Aware network (SAA) to extract the multi-frame correlations more effectively. To be more specific, our Scale-and-Attention Aware Module (SAAM) applies different numbers of feature extraction layers to acquire multi-scale feature representations from separate neighboring frame streams. Considering that the closer the neighboring frame is to the center frame, the easier the motion estimation and thus the more accurate are the warped frames that can be obtained, we design a center-oriented fusion manner to apply more attention to frame streams that are close to the center. Furthermore, we propose a Criss-Cross Channel Attention Module ($C^{3}AM$) to extract spatial and channel attention of each feature extraction stream more effectively and efficiently.

The main contributions of this work can be summarized as follows:

- We propose a Scale-and-Attention Aware network (SAA) for video SR. Experiments on several public datasets demonstrate the effectiveness of our SAA networks.
- We propose Scale-and-Attention Aware Module (SAAM) to fuse and extract features in multi-frame streams. This module design methodology not only extracts multi-scale feature information but also obtains feature correlations explicitly and effectively, thanks to the center-oriented fusion manner and the spatial–channel attention extraction mechanism in $C^{3}AM$.
- We propose the Criss-Cross Channel Attention Module ($C^{3}AM$) to extract spatial and channel attention information inherent in multi-frame stream features. With the aid of criss-cross non-local implementation, spatial correlations can be obtained both efficiently and effectively. The channel attention mechanism further helps the module to acquire attention weights in the channel dimension for even better feature representations. Both criss-cross non-local and channel attention mechanisms help extract feature correlations effectively with negligible computation overhead and extra parameters compared to vanilla non-local implementation.

## 2. Related Works

As a basic problem, SR has been researched for decades. We focus on deep learning SR methods and the flourishing attention mechanisms in recent years. Besides, we mainly introduce several deep learning based motion estimation methods that are fairly relevant to our work due to the space limitation. A more comprehensive review about VSR can refer to the recent work [11].

### 2.1. Deep Single Image SR

Since Dong et al. [12] pioneer the work in deep learning methods for SISR with a three-layer CNN network, plenty of CNN-based SISR methods [13,14,15,16,17,18,19,20,21] have been proposed to handle the challenging ill-posed problem in a data-driven manner. The research in this field has focused on amplification layers or manners [15,22,23], residual or dense blocks utilization [24,25], deeper networks [18,19,24], adversarial methods [16,26], and so forth. Some methods also explore intra-image discipline and recover more HR image details by back-projection [27] and higher-order attention [20,28] in recent years.

### 2.2. Deep Video SR

Liao et al. [29] made the first endeavour to embrace deep learning in video SR. SR draft image patch candidates are generated with adjusted flow-based motion estimation, and are then fed into a shallow CNN network to combine the draft-ensembles in a learning style. Kapperler et al. [7] also compensated for the motion of consecutive LR frames and then concatenated before the CNN reconstruction. Liu et al. [30] designed a temporal adaptive network to fuse multi-inference branches with different time spans. Caballero et al. [8] compensated for the motion of neighbor frames with an end-to-end spatial transformer network and combined the compensated frames in a slow-fusion manner. Tao et al. [2] also followed the ME and MC paradigm for neighbor frames’ alignment. They innovate the warping operations by proposing forward warping and the SPMC layer in their work. Different from preceding methods, they process warped HR frames with encoder–decoder architecture and ConvLSTM layers in a recurrent manner for better scalability. Sajjadi et al. [3] warp the previously inferred HR estimate with the upsampled estimated optical flow, then combine the warped HR estimate with the current LR center frame to predict the final current HR frame. Later, Chan et al. [31] designed an efficient VSR, named BasicVSR, by reconstructing some of the most essential components for VSR. Other methods focus on designing more efficient VSR methods by knowledge distillation [6] and neural architecture search [32]. All the above methods explicitly compensate for the motion between neighbor and center frames with usually flow-based warping operations. These methods can accomplish motion compensation directly with the assistance of estimated motion. However, motion estimation tends to be inaccurate and the resultant warping errors and artifacts can do non-negligible damage to the final reconstruction results, even with slow-fusion techniques or learned temporal dynamics. Consequently, different attentions should be paid to warped frames with different warping accuracies, and further feature correlations should be focused on posterior fusion operations, which will be illustrated in our main method.

Recently, many efforts have been devoted to motion compensation implicitly without the aid of optical flow. Jo et al. [33] design a share-weight CNN network to generate dynamic upsampling filters and HR residual images using the LR frames consecutively. Then, the center frame is filtered by the generated dynamic filters and added by the estimated HR residual image to obtain the final HR prediction. Li et al. [10] adopt depth-wise separable 3D convolutions to directly fuse the multiple LR frames. Instead of stacking or warping the LR frames, Haris et al. combine the multi-frame sources in an iterative refinement framework with a back-projection technique to fuse the multi-stage prediction outputs. Yi et al. [4] introduce the non-local networks [34] to replace the complex ME&MC operations to do implicit warping for LR frames. They further propose a progressive fusion strategy to fuse the implicitly compensated LR features progressively. These works all make efforts to bypass the complex ME&MC pre-processing paradigm and explore various fusion methods to combine the multi-frame features together. However, they neglect the different importance of different LR frames with different temporal spans from the center frame. Besides, correlations inherent in the features have not been utilized sufficiently and thoroughly. Considering the limitations of the previous methods, we propose Scale-and-Attention Aware Networks (SAA) to apply different attention to separate LR frame streams and further extract spatial and channel feature correlations explicitly with the proposed $C^{3}AM$ modules.

### 2.3. Attention Mechanism

Akin to the human visualization system, deep neural networks can benefit from attention to salient areas of features. To utilize the intra-attention on different positions of a single sequence or feature, a self-attention mechanism [35] is proposed in natural language processing and achieves promising performance gains in relevant fields. Inspired by the self-attention in NLP and non-local means [36] in traditional image processing, Wang et al. [34] propose non-local neural networks to apply non-local operations to acquire spatial attention of intermediate features in video classification task. Yi et al. [4] apply the non-local mechanism in video SR task to avoid the complex ME&MC procedures. Despite the considerable progress it achieves, non-local operations in the whole space of the feature leads to a considerable computation burden, which cannot be neglected when the spatial size of the feature is not small or the non-local layers are consecutively used in the networks. To extract spatial correlations in a more effective and efficient way, Huang et al. [37] propose Criss-Cross Network (CCNet) to collect the non-local information of a specific position on its criss-cross path. With two recurrent criss-cross layers that share weights, the criss-cross attention mechanism can finally provide non-local attention for each position with the whole spatial feature information. Apart from modelling the spatial correlations of deep features, Hu et al. [38] exploit channel-wise correlations with the channel attention mechanism, which builds interdependencies between channels and eventually enhances the representation capability of the network.

## 3. Scale-and-Attention Aware Networks (SAA)

Given a low-resolution frames sequence $\left\{I_{LR}^{t-N},\ldots ,I_{LR}^{t},\ldots ,I_{LR}^{t+N}\right\}$, where *t* denotes the current time step and *N* denotes the time span radius of the input *LR* sequence, the aim of our SAA is to recover the $t^{th}$ high-resolution frame $I_{HR}^{t}$.

We will first take a full glance at the overall structure of our proposed SAA networks. Then, we illustrate the basic unit of SAA, that is, Scale-and-Attention Aware Module (SAAM), including the Multi-Scale Feature Extraction and the Center-oriented Fusion (MFE-CF) mechanism and Criss-Cross Channel Attention Mechanism ($C^{3}AM$). As the key mechanism in the feature extraction and fusion module, the proposed $C^{3}AM$ will be elaborately illustrated. Implementation details and further comparisons with other relevant methods will be exhibited at the end of this part.

### 3.1. Overall Network Architecture

**Figure 1:**
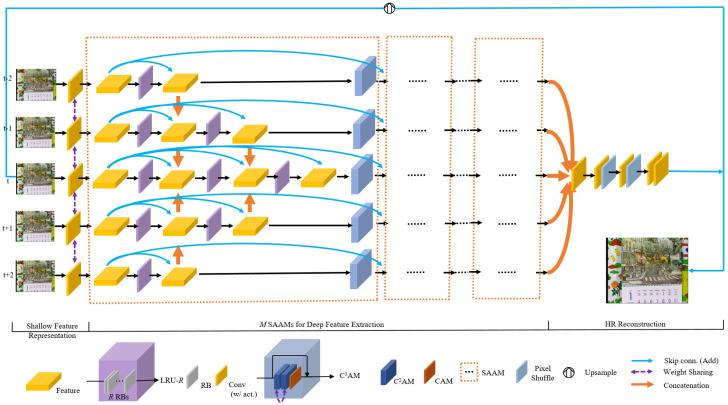
Overall Architecture of our proposed Scale-and-Attention Aware Networks (SAA) and its sub-modules.

As shown in Figure 1, our SAAN is composed of four main parts: shallow feature representation, deep feature extraction composed of *M* Scale-and-Attention Aware Modules (SAAMs), and HR reconstruction.

Given the consecutive *T* input *LR* frames $\left\{I_{LR}^{t+i}|i=N,\ldots ,0,\ldots ,N\right\}$, where T=2N+1, we firstly extract feature embedding information of each input *LR* frame through a sharing-weight shallow feature representation part $H_{FR}$:

(1) $$F_{0}^{t+i}=H_{FR}\left(I_{LR}^{t+i}\right),$$

where $F_{0}^{t+i}$ denote the embedding representation of ${\left(t+i\right)}^{th}$ *LR* frame $I_{LR}^{t+i}$, and $H_{FR}\cdot$ denotes the shallow feature representation module. To be more specific, the separate input *LR* frames are first convolved by a nonlinear mapping layer, and are then further represented by five consecutive residual blocks (each consists of two convolution layers with skip connection).

To further extract feature information and acquire the deep feature correlations, the extracted features are further processed by *M* consecutive Scale-and-Attention Aware Modules (SAAMs) to obtain the deep feature representations:

(2) $$F_{EX}=H_{FE}\left(\left\{F_{0}^{t+i}\right\}\right),$$

where $H_{FE}\left(\cdot \right)$ represents *M* consecutive Scale-and-Attention Aware Modules (SAAMs). With the very deep feature extraction sub-module and the proposed Criss-Cross Channel Attention mechanism embedded in it, the network can acquire a large enough receptive field and fully utilize the spatial and channel correlations inherent in the deep features.

Afterwards, the extracted deep features $F_{EX}$ is upsampled by two consecutive pixel shuffle [23] layers to be mapped onto the *HR* feature space:

(3) $$F_{↑}=H_{↑}\left(F_{EX}\right),$$

where $H_{↑}$ and $F_{↑}\left(\cdot \right)$ represent the upsampling layers and upsampled deep features, respectively. Afterwards, the upsampled features $F_{↑}$ are further reconstructed by two non-linear mapping layers in the *HR* space to obtain the *SR* frame. For better gradient propagation and feature delivery, we adopt the residual learning scheme and the reconstructed *HR* residual frame is further added by the bilinearly upsampled center frame:

(4) $$I_{SR}^{t}=H_{R}\left(F_{↑}\right)+U_{↑}\left(I_{LR}^{t}\right)=H_{SAA}\left(\left\{I_{LR}^{t+i}\right\}\right),$$

where $I_{SR}^{t}$, $I_{LR}^{t}$, $H_{R}$ and $U_{↑}$ denote the final output *HR* center frame, the input *LR* center frame, the *HR* reconstruction non-linear mapping layers and the bilinear upsampling operations, respectively.

To harvest the merits of both the robustness towards outliers when compared to the MSE loss and the differentiability near zero, we adopt Charbonnier loss for optimization. The goal of SAA is to optimize the Charnonnier loss function:

(5) $$L\left(\Theta \right)=\frac{1}{B}\sum_{i=1}^{B}\sqrt{\left|\right|H_{SAA}\left(\left\{I_{LR}^{t+i}\right\}\right)-I_{HR}^{t}{\left|\right|}_{2}^{2}+\epsilon^{2}},$$

where Θ denotes the learnable parameters of SAA, *B* denotes the numbers of samples, $I_{HR}^{t}$ denotes the ground truth *HR* center frame, and ϵ is a small constant. (We empirically set $\epsilon =10^{-6}$ for all our experiments.)

### 3.2. Scale-and-Attention Aware Module (SAAM)

As the backbone structure of the proposed SAA networks, the multiple consecutive Scale-and-Attention Modules (SAAMs) for deep feature extraction plays an essential role for the overall video super-resolution framework. As shown in Figure 1, each SAAM adopts a multi-branch designing manner, and the number of branches coincides with the input frames. The SAAMs take as input the multi-frame shallow feature representations, extract and fuse the features in a unified and progressive way, with feature spatial and channel correlations fully exploited and temporal correlations excavated appropriately and automatically. Given the output multi-frame features of ${\left(j-1\right)}^{th}$ SAAM, denoted as $\left\{F_{{SAAM}_{j-1}}^{t+i}\right\}$, the $j^{th}$ SAAM extracts the multi-frame features and obtains multi-branch features for the next procedure:

(6) $$\left\{F_{{SAAM}_{j}}^{t+i}\right\}=H_{{SAAM}_{j}}\left(\left\{F_{{SAAM}_{j-1}}^{t+i}\right\}\right),$$

where $H_{{SAAM}_{j}}$ and $\left\{F_{{SAAM}_{j}}^{t+i}\right\}$ denote the $j^{th}$ SAAM module and its output features, respectively.

**Multi-Scale Feature Extraction and Center-oriented Fusion (MFE-CF).** Specifically, SAAM processes the features parallelly and takes a center-oriented fusion direction for multi-frame feature utilization and fusion. Furthermore, out of the consideration of multi-frame discrepancies of significance for center frame super resolution, we design a multi-scale processing mechanism for multiple feature branches. To be more specific, different numbers of consecutive convolution layers are adopted on multiple branches and the more the branch comes near the center frame feature extracting branch, the more feature extraction operations are laid aside for the purpose of not only the wider receptive field but the more accurate and refined deep feature representations. To further improve the effect of feature extraction and fusion, we adopt the local residual units (LRUs) as the basic units of feature extraction and fusion in each temporal stream. As shown in the purple modules in Figure 1, LRU-*R* represents a local residual unit composed of *R* consecutive residual blocks. With the design methodology of multlocal residual unit-scale feature extraction and center-oriented fusion, multi-frame features are extracted and fused with different scales and non-linear mapping layers give expression to the unequal status for deep features on different temporal branches. We will show the necessity and significant performance gain through the proposed multi-frame features processing pattern of multi-scale feature extraction and center-oriented fusion with ablation studies in Section 4.4.

**Criss-Cross Channel Attention Module ($C^{3}AM$).** After the multi-scale feature extraction and center-oriented fusion stage in each Scale-and-Attention Aware Module (SAAM), the multi-features $\left\{F_{{MFE}_{j}}^{t+i}\right\}$ with temporal correlations and multi-scale information inherent in it have been obtained. However, the correlations in the spatial and channel dimension of extracted features in each temporal branch have not been taken into full consideration. To better exploit the non-local correlations in features and acquire possible information gain through spatially similar positions, and refine channel-wise inter-dependencies in the meantime, we design a Criss-Cross Channel Attention Module ($C^{3}AM$) for better feature correlation exploitation and utilization.

**Share-Source Residuals (SSR) and Residuals between SAAMs.** As shown in Figure 1, to avoid gradient vanishing or gradient diffusion in the training procedure, and promote the reuse of features, we adopt residual connections in SAAMs. Specifically, we adopt share-source residuals, which connect beginning input features and features processed by each LRU in each temporal branch. Besides, we connect the input features of neighboring SAAMs in each temporal stream to further facilitate gradient propagation and improve feature information interchange between consecutive SAAMs. We use element-wise adding operation for skip connections.

### 3.3. Criss-Cross Channel Attention Module ($C^{3}AM$)

The proposed SAA acquires spatial and channel correlations of the feature maps of each temporal branch in SAA through the proposed Criss-Cross Channel Attention Module ($C^{3}AM$). Given the features in the ${\left(t+i\right)}^{th}$ temporal branch after the multi-scale feature extraction and the center-oriented fusion procedure of SAAM, which is denoted as $F_{MS}^{t+i}$, the $C^{3}AM$ module aims to acquire the multi-frame features with both spatial and temporal correlations.

**Criss-Cross Attention.** A possible solution to acquire spatial attention is the non-local module [34], which calculates spatial correlations for one single position with every position available in the full spatial space. However, this method requires memory and computation usage with the complexity of $O({\left(H\times W\right)}^{2})$ and is therefore impractical for input features with high spatial sizes. Besides, this method of directly computing spatial correlations from all spatial positions towards the current target position tends to produce inaccurate attention outputs, since the difficulty of modelling all the spatial similarities for the target position increases radically, especially when the candidate positions become excessive for features with big spatial sizes.

Based on the considerations above, we adopt the criss-cross attention mechanism [37] for spatial correlation acquisition. The criss-cross attention exploits the spatial correlations for the target position with only the positions that are on the criss-cross path of target position, that is, the same row or column. The correlations from other positions towards the target position can be further obtained via two recurrent criss-cross attention modules, which adopt a weight-sharing manner. The criss-cross attention mechanism costs the complexity O((H+W−1)·(H×W)) of memory and computation in each recurrence, making it relatively efficient to be embedded into the multi-frame branches for better spatial correlation extraction. Besides, with the sharply decreased candidate position for the correlation computation of each target position, the attention modelling for target position becomes easier and more reliable. As a consequence, the non-local similarities of multi-frame features are fully exploited by the recurrent criss-cross attention mechanism both effectively and efficiently.

**Channel Attention.** The spatial correlations are obtained by the recurrent cross-cross attention mechanism with a relatively low computation cost compared with vanilla non-local implementations. However, the channel-wise responses are not adaptively recalibrated with the spatial attention mechanism alone. To further exploit the channel relationship inherent in the multi-frame features, we fuse the channel-wise attention to the criss-cross based spatial correlations by stacking a channel attention module after the recurrent criss-cross attention module. To be more specific, given the features with spatial correlations extracted from the recurrent criss-cross attention mechanism, that is, $F_{CC}$, the global embedding information is firstly squeezed via an average pooling operation on the full spatial dimension. The channel-wise correlations is further exploited by an excitation process with two consecutive fully-connected layers for adaptive recalibration. This channel correlation information is further multiplied by the output features of criss-cross attention module $F_{CC}$ with spatial attention correlations to obtain the features $F_{C^{3}A}$ with both spatial and channel attention. For the consideration of better gradient propagation and feature information interaction, the extracted spatial channel features $F_{C^{3}A}$ is eventually added by the input features $F_{MS}^{t+i}$ to obtain the final output features $F_{C^{3}A}^{t+i}$ of $C^{3}AM$ in the ${\left(t+i\right)}^{th}$ temporal branch.

After the consecutive Criss-Cross Attention and Channel Attention mechanism, the spatial and channel correlations of multi-frame features are fully exploited, and are provided for the next SAA stage for further feature extraction.

### 3.4. Implementation Details

We use M=10 SAAs in the SAAM, and F=7 multi-frame branches for a better performance/complexity trade-off. Number of residual blocks *R* in each local residual unit LRU-*R* is set as 3. Detailed experiments and analyses for connections between model configuration and performance, and the trade-off between the two are elaborated in Section 4.5. Unless otherwise stated, 3×3 kernel convolution with C=64 filters is adopted in all layers. The recurrence times Rec of the criss-cross module is set as 2 with the least cost for obtaining the global non-local correlations. We employ the reduction ratio red=16 for the excitation process in the channel attention mechanism.

### 3.5. Discussions

**Differences with VSRNet [4].** VSRNet explores various ways to utilize multi-frame information, including concatenating the frames directly before the convolutional layers, or imposing the concatenation procedure after a certain layer. However, this method does not take the multi-frame correlations into consideration explicitly and the direct fusion manner tends to fail in fully taking advantage of the information gains in neighboring frames without the effective correlation learning and utilization process.

**Differences with PFNL [7].** PFNL [4] also adopts a progressive manner for multi-frame fusion but our proposed SAA networks differentiate on the summarized aspects. The first one is that all the multi-frame branches in each PFNL’s fusion block act as the equal role, follows the same “separable-fusion-separable” manner to acquire the fused features. In contrast, we take the different significance of each temporal branch into consideration and employ different numbers of non-linear mapping layers to impose a higher significance level on the branches closer to the center frame branch, considering that the feature information on these branches is more accurate and is relatively easier for the super-resolution on center frame, since the further one frame goes away from the center in the temporal scale, the relative displacement between them tends to be more complex and thus the difficulty for the network to transfer extra information from neighbor frame features to the center ones increases rapidly. Besides, thanks to the more non-linear mapping layers, the center and near-center branches acquire larger receptive fields, which make each SAAM module more sensitive to the minor changes in wider spatial range on these branches, helping the network become aware of the information that is more accurate and easier to acquire for center frame super-resolution. Furthermore, we take spatial and channel attention into consideration, and design a Criss-Cross Channel Attention Module ($C^{3}AM$) for spatial and channel correlations extraction and utilization on each temporal branch, with minor computation overload increased from the no-attention baseline, compared with the original non-local operations or the adaptively changed version in PFNL’s non-local alignment module. More specifically, the criss-cross attention mechanism adopted in our $C^{3}AM$ needs the complexity of O((H+W−1)·(H×W)) of computation (as analysed in Section 3.3), while the original non-local implementation needs $O({\left(H\times W\right)}^{2})$, and the adaptive version in PFNL takes $O({\left(\frac{\left(H\times W\right)}{r}\right)}^{2})$ computations (*r* denotes the reduction ratio in both width and height dimension, which is introduced for performance/complexity trade-off). In addition, the channel attention mechanism in our $C^{3}AM$ module takes O((H×W)) computation, which can be ignored when compared with spatial correlation calculations. All the complexity analyses above assume that the lengths of both temporal and channel dimensions are constants when compared with the spatial dimensions.

## 4. Experiments

### 4.1. Datasets

**Vimeo-90K Dataset.** The Vimeo-90K dataset [39] is a large-scale public video dataset, consisting of 90,000 high-quality video clips. The Vimeo-90K dataset contains ample and real scenes and various complexities of motion patterns, and rare degradations or noises. As a consequence, the Vimeo-90K dataset is widely used in various video restoration tasks, and is one of the most recognized public dataset in video super-resolution. In video SR, a subset of the Vimeo-90K dataset, which consists of 64,612 video clips, is usually selected as the training dataset, denoted as Vimeo-Train. Another non-overlapping subset with 7824 video clips is divided as a testing dataset, denoted the Vimeo-Test. In the Vimeo-90K dataset for Video SR, each video clip contains 7 frames, and the VSR model only super-resolve the center frame for qualitative and qualitative evaluations. The resolution of all ground truth video frames is 448×256. As other methods, standard bicubic downsampling kernel is adopted to generate LR video frames from HR ground truth frames for both training and testing phases.

**Vid4 Dataset.** The Vid4 dataset [40] is a frequently-used public testing dataset for evaluations of video SR methods. The Vid4 dataset is composed of four video clips named *walk*, *foliage*, *city* and *calendar*. A bicubic downsampling kernel is used for the Vid4 dataset for performance evaluation.

**SPMCS Dataset.** The SPMCS dataset [2] is another common dataset used for VSR methods evaluations. This dataset consists of 30 video clips, each with 31 frames. The ground truth scenes are noise-free and contains abundant high-frequency details. The ground truth videos are with 540×960 resolution, which are bicubicly downsampled from original 1080p videos captured with high-end camera. LR video frames with resolution 135×240 are further obtained from the growth videos with bicubic downsampling. To compare the detailed evaluation performance of each video clip, we adopt 11 typical video clips for quantitative evaluation.

### 4.2. Configurations

All PSNR and SSIM evaluations are conducted on the Y channel of YCbCr space or on RGB color space in all our experiments. During the training procedure, we use batch size 32 and patch size 64×64 and employ random flipping data augmentation for input frames. The Charbonnier loss function is adopted with ϵ=1e−6 and we choose the Adam optimizer [41] with $\beta_{1}=0.9$ and $\beta_{2}=0.999$. The initial learning rate is set as $4\times 10^{-4}$ and we adopt a cosine annealing strategy to decay the learning rate. We train our SAA model for $1.8\times 10^{6}$ iterations for convergence. We implement our method on Pytorch framework [42] and train the model on eight Tesla P40 GPUs.

For the experiments for ablation studies in Section 4.4 and module analyses in Section 4.5, we use a low-cost configuration and adopt batch size 32 and patch size 32×32, and train the model for $1.5\times 10^{5}$ iterations.

### 4.3. Comparison with State-of-the-Art Methods

We compare our proposed SAA networks with various video SR state-of-the-arts, including SISR methods such as RCAN [18], and VSR methods such as: DeepSR [29], BayesSR [43], VESPCN [8], DRVSR [2], TOFlow [39], FRVSR [3], DUF [33], RBPN [9] and TDAN [44], and so forth. Since severe boundary artifacts appear in results of DUF [33], we crop eight boundary pixels of the results of DUF [33] before quantitative evaluation. We do not impose cropping boundary operations for any other methods. To further improve the performance of our SAA, we adopt self-ensemble strategy in the testing phase, denoted as “SAA+” in quantitative results tables.

**Figure 2:**
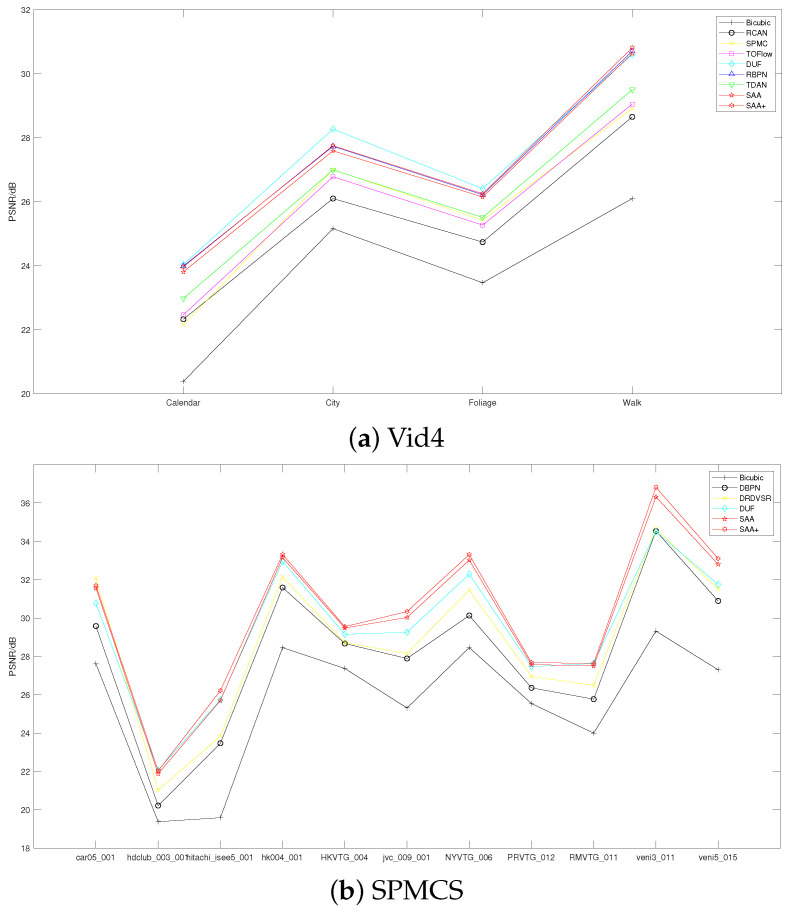
PSNR curves of our proposed SAA and other VSR methods on (**a**) Vid4 and (**b**) SPMCS datasets.

The quantitative evaluation results on Vimeo-Test, Vid4, and SPMCS datasets are listed in Table 1, Table 2 and Table 3, respectively. As shown in Table 1 and Table 3, SAA outperforms other state-of-the-art methods in super-resolution by a large margin on PSNR (dB)/SSIM metric in the Vimeo-Test and SPMCS datasets. In Table 2, SAA surpasses other methods including the most recent state-of-the-art RBPN [9] and TDAN [44] but is inferior to to DUF [33] by 0.13 dB on average PSNR. However, DUF [33] uses a non-public training dataset, which coincides more with the Vid4 dataset on data distribution. As a whole, our SAA performs much better than other state-of-the-arts on quantitative evaluation, which demonstrates the effectiveness of the proposed method. To have an intuitive understanding of our SAA with other SR methods, we give PSNR plots of different SR methods in Figure 2, from which we can see that our SAA obtains the best results in most cases.

**Figure 3:**
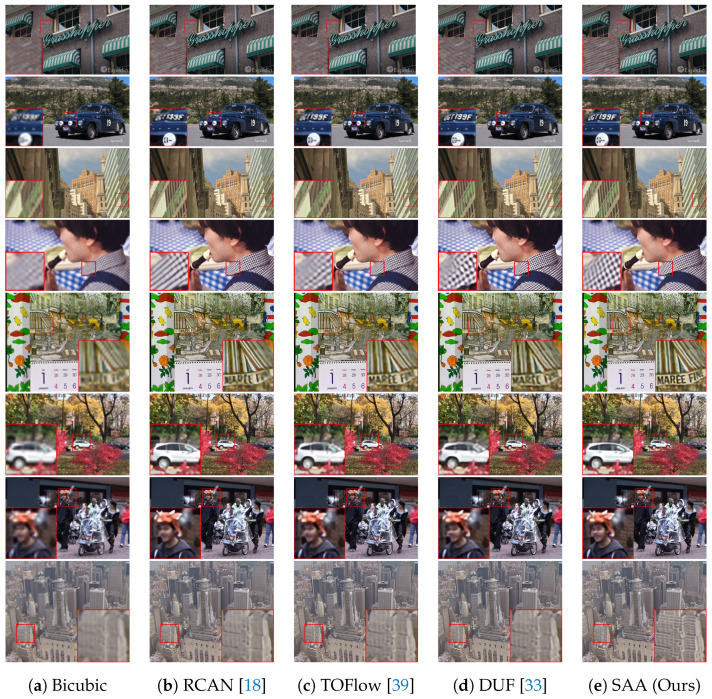
Visual comparison of our proposed SAA and other methods on different datasets.

Besides, to reveal the superiority of SAA over other methods on visual effects, we compare the results of SAA and other methods on several representative SR frame results selected from clips with fairly rich details. The visual results are shown in Figure 3. As shown in the upper scene of Figure 3, TOFlow [39] and DUF [33] can hardly recover high-frequency details and fine textures of the brick wall. Textures direction predicting errors and even severe artifacts appear in the SR result of DUF [33]. Compared with RCAN [19], TOFlow [39], DUF [33] and RBPN [9], our proposed SAA can reconstruct the brick wall with sharp edges and correct directions. In general, much finer details and textures of the brick wall can be recovered by our method. TOF [39] and DUF [33] failed to reconstruct the sharp edge details. RCAN [19] and RBPN [9] can recover some structures, but predicts wrong edge and structure details. In contrast, SAA can recover more image details with correct structures and fine details, which proves the effectiveness and robustness of the proposed method.

As a consequence, the alphabets and numbers can hardly be recognized from the SR result generated by RCAN [18]. TOFlow [39] can only recover partial information of alphabets and numbers, while inaccurate predicted regions exists. The problem of distortions and the deficiency of high-frequency information need to be solved. DUF [33] can recover the four relatively easy alphabets or numbers in the right side, but fails for the left two. It can also be observed that DUF [33] cannot rebuild the details on the white round ornaments between the headlights. In contrast, SAA recovers the relatively accurate structures and details of the alphabets and numbers on the licence plate and the details on the ornaments. The results on the other three frames in Figure 3 also coincide with the aforementioned descriptions. Specifically, SAA can recover fine structures of the city building, the textures in the plaid shirt and the details of the person’s mouth, while other methods generate fairly defective results. These qualitative results and comparisons show that SAA is able to generate super-resolved videos with fine details and pleasant visualizations, which supports the effectiveness of our method. This is because our SAA can effectively utilize and fuse the extracted features with the beneficial correlations provided by the joint spatial and channel attention mechanism, leading to much finer features with spatial and channel correlation information inherent in the features.

### 4.4. Ablation Study

#### 4.4.1. Multi-Scale Feature Extraction and Center-oriented Fusion (MFE-CF) and Criss-Cross Channel Attention Module ($C^{3}AM$)

As discussed in Section 3.2, Multi-Scale Feature Extraction and Center-oriented Fusion (MFE-CF) and Criss-Cross Channel Attention Module ($C^{3}AM$) are the two key designs of our Scale-and-Attention Aware Module (SAAM). To verify the performance gain brought by the two proposed mechanisms, we conduct experiments on the networks with and without these mechanisms, and the results are listed in Table 4.

As shown in Table 4, $S_{a}$ refers to the baseline model, which removes both MFE-CF and $C^{3}AM$ from each SAAM of our proposed SAA networks. $S_{b}$ model, which is designed to validate the influence of MFE-CF, represents the model that introduces the MFE-CF mechanism to the baseline model $S_{a}$. Correspondingly, $S_{c}$ model explores the effectiveness of $C^{3}AM$, with extra $C^{3}AM$ mechanism compared with the baseline $S_{a}$ model. $S_{d}$ denotes the adopted model with both MFE-CF and $C^{3}AM$ mechanisms. As shown in Table 4, $S_{b}$ outperforms $S_{a}$ by 0.23 dB and 0.19 dB on Y channel of YCbCr color space and RGB channels, respectively. This is because the MFE-CF mechanism imposes different numbers of convolutional layers for temporal branches with different time spans apart from center branch, thus leading to larger receptive fields for relatively near branches. In the meantime, the center-oriented fusion manner merges marginal temporal branches progressively for stable and efficient feature fusion. As a consequence, the SAAM is able to utilize the most important features extracted from the center frame and near neighbor frames, leading to much better feature utilization effectiveness and fairly remarkable performance gain. Similarly, $S_{c}$ model surpasses baseline model $S_{a}$ evidently with the assistance of $C^{3}AM$ mechanism. The introduce of $C^{3}AM$ leads to 0.29 dB and 0.22 dB PSNR gain on Y and RGB channels, respectively. The performance gain is obtained via the spatial and channel correlation information of features in each temporal stream. These correlations are calculated explicitly by $C^{3}AM$, and are then applied to the latter feature extraction and reconstruction. Therefore, spatial and channel attention is introduced to feature representations, enabling the model to reconstruct HR frames with more spatial correlation information about neighboring pixels and channel correlations, and thus, more high-frequency details. Eventually, when both MFE-CF and $C^{3}AM$ mechanisms are introduced, the finally adopted $S_{d}$ model can take the merits of both effective feature extraction and fusion manner and joint spatial and channel correlations. Therefore, the model capability of feature representation is enhanced, leading to superiority in model performance. Taking advantage of the MFE-CF and $C^{3}AM$ mechanisms, the eventually adopted $S_{d}$ model significantly outperforms the $S_{a}$ baseline by 0.36 dB and 0.31 dB in Y and RGB channels, respectively, as shown in Table 4, which demonstrates the effectiveness of both mechanisms.

#### 4.4.2. Share-Source Residuals in SAAMs and Residuals between Adjacent SAAMs

As elaborated in Section 3.2, to facilitate gradient propagation and promote effective utilization of features, each SAAM adopts share-source residual (SSR) connections. Specifically, as shown in Figure 1, on each temporal stream inside an SAAM, each local residual unit (LRU) takes the beginning features of the stream as an input to promote gradient propagation and feature reuse. Furthermore, we also connect features from the input of adjacent SAAMs with skip connections on each corresponding temporal stream, as shown in Figure 1, to further facilitate training optimization and feature utilization. We use element-wise adding for the aforementioned residuals or skip connections, therefore *no* extra parameters are needed. To verify the effectiveness of share-source residuals (SSR) and residuals between adjacent SAAMs, we conduct ablation studies towards these two types of residuals. The results are shown in Table 5. As shown in Table 5, $S_{e}$ represents the model which removes both SSRs in each SAAM and residuals between SAAMs from the baseline model $S_{d}$. $S_{f}$ represents the model with residuals between SAAMs, which is designed to verify the effect of corresponding residuals. The $S_{g}$ model introduces share-source residuals to each SAAM, aiming to prove the model capability promotion brought by SSRs. Finally, $S_{d}$ is the adopted model with both residual connections.

It can be concluded from Table 5 that $S_{f}$ model with residuals between SAAMs surpasses the baseline model $S_{e}$ by 0.22 dB and 0.31 dB in Y and RGB channels, respectively, while $S_{g}$ models with SSRs outperforms the baseline $S_{e}$ model by 0.22 dB and 0.38 dB in Y and RGB channels, respectively. Furthermore, the eventually adopted $S_{d}$ model outperforms the $S_{e}$ baseline by an even larger margin. These quantitative results show that both types of residuals benefit the performance of models, which demonstrate the necessity of both types of residuals. This is because these adopted residuals can promote gradient propagation and help the model to effectively utilize shallow features with more detailed information, which is significantly important for VSR to achieve the goal of recovering high-resolution video frames with high-frequency details.

### 4.5. Model Analyses Experiments

To analyze the effect of model configurations for SAA towards model performance, we design experiments to analyze four model configuration hyper-parameters. The configurations are the number of SAAMs (*M*), the number of temporal streams/input frames *F*, model channels and the number of residual blocks in each local residual units (LRU). The results are shown in Table 6, Table 7, Table 8 and Table 9.

#### 4.5.1. Influence of Number of SAAMs (*M*)

One advantage of our proposed SAA networks is that the backbone structure of our SAA networks is simply stacked by multiple SAAM modules, the number of which can be flexibly and conveniently adjusted for different performance/complexity trade-offs in various application scenarios. We thus perform experiments on the performance and model complexity variation brought by different numbers of SAAMs. As shown in Table 6, within limits, model performance can be promoted by increasing the numbers of SAAMs (*M*), since model capacity builds up during the process. However, model performance increases more slowly when *M* gets larger, while model complexity increases linearly. Therefore, we choose M=10 for a better performance/complexity trade-off as stated in Section 3.4.

#### 4.5.2. Influence of Number of Temporal Branches (*F*)

As shown in Figure 1, each SAAM module in SAA networks consists of multiple temporal branches and the number of temporal branches (*F*) coincides with the number of input frames. We conduct experiments to explore the influence of various numbers of temporal branches. As shown in Table 7, changing the number of temporal branches/input frames (*F*) makes a significant difference to model performance. To be more specific, PSNR in the Y channel of YCbCr color space increases 0.38 dB, 0.48 dB and 0.27 dB when changing temporal branches from 1 to 3, from 3 to 5 and from 5 to 7, respectively. PSNR in the RGB channels increases accordingly. We fix the number of temporal branches (*F*) to seven for the best model performance, since each video clip in Vimeo-90K consists of seven consecutive frames.

#### 4.5.3. Influence of Number of Channels (*C*)

To analyze the influence of model channels *C*, we fix other configurations and adjust the value of *C*. As shown in Table 8, when channels are enlarged from 16 to 32, from 32 to 64 and from 64 to 128, PSNR in the Y channel of YCbCr color space increases by 0.48 dB, 0.42 dB and 0.25 dB respectively, and PSNR in RGB channels increases correspondingly. Given that, when doubling channels, the number of model parameters is quadrupled, we choose C=64 in the eventual model described in Section 3.4 for better performance/complexity trade-off.

#### 4.5.4. Influence of Number of Residual Blocks (*R*) in Local Residual Unit (LRU)

In the end, we design several experiments to analyze the influence of the number of residual blocks (*R*) in a local residual unit (LRU) in SAAMs. As shown in Table 9, when we increase *R* from 1 to 2, from 2 to 3 and from 3 to 4, PSNR in the Y channel increases by 0.16 dB, 0.12 dB and 0.06 dB, respectively. As with the other configurations, the variation trend of RGB PSNR coincides with PSNR in the Y channel. This implies by stacking the number of residual blocks in the basic local residual units, the feature extraction and representation capability of SAA is strengthened. However, when we increase the value of *R*, the model complexity also increases rapidly. Besides, when *R* is equal to or greater than 3, the model performance promotion becomes insignificant by enlarging *R*. Therefore, we choose R=3 in each local residual unit (LRU) for the eventually determined SAA model in Section 3.4.

## 5. Conclusions

In this work, we propose novel Scale-and-Attention Aware Networks (SAA) that utilize multi-scale feature information and feature correlations for video SR. Specifically, Scale-and-Attention Aware Modules (SAAMs) adopt a multi-scale manner and impose adaptive attention on different streams with different time spans apart from the center frame for better multi-frame feature extraction and fusion. The Criss-Cross Channel Attention Module ($C^{3}AM$) is designed to explicitly exploit the spatial and channel attention of multi-stream features. Furthermore, we adopt multi-scale feature extraction and center-oriented fusion (MFE-CF) to promote the use and fusion of multi-stream information. Compared with other SR methods, our method obtains average PSNR gains by 0.2 dB on the SPMCS dataset. Extensive experiments on public video SR datasets demonstrate the superiority of our method over other state-of-the-art methods.

## Figures and Tables

**Table 1:** Quantitative results of PSNR(dB)/SSIM of our proposed SAA networks compared with Bicubic and other state-of-the-art methods on **Vimeo-90K**. The two best-performing methods are marked in **bold** and are underlined respectively.

Methods	PSNR (dB)/SSIM (RGB)	PSNR (dB)/SSIM (Y)
Bicubic	29.79/0.8483	31.32/0.8684
RCAN [18]	33.61/0.9101	35.35/0.9251
DeepSR [29]	25.55/0.8498	-/-
BayesSR [43]	24.64/0.8205	-/-
TOFlow [39]	33.08/0.9054	34.83/0.9220
DUF [33]	34.33/0.9227	36.37/0.9387
RBPN [9]	-/-	37.07/0.9435
SAA (Ours)	35.22/0.9310	37.00/0.9432
SAA+ (Ours)	**35.44**/**0.9329**	**37.24**/**0.9448**

**Table 2:** Quantitative results of PSNR(dB)/SSIM of our proposed SAA networks compared with Bicubic and other state-of-the-art methods on **Vid4**. The two best-performing methods are marked in **bold** and underlined, respectively.

Methods	Calendar (Y)	City (Y)	Foliage (Y)	Walk (Y)	Average (Y)
Bicubic	20.39/0.5720	25.16/0.6028	23.47/0.5666	26.10/0.7974	23.78/0.6347
RCAN [18]	22.33/0.7254	26.10/0.6960	24.74/0.6647	28.65/0.8719	25.46/0.7395
VESPCN [8]	-/-	-/-	-/-	-/-	25.35/0.7557
SPMC [2]	22.16/0.7465	27.00/0.7573	25.43/0.7208	28.91/0.8761	25.88/0.7752
TOFlow [39]	22.47/0.7318	26.78/0.7403	25.27/0.7092	29.05/0.8790	25.89/0.7651
FRVSR [3]	-/-	-/-	-/-	-/-	26.69/0.8220
DUF [33]	**24.04**/**0.8110**	**28.27**/**0.8313**	**26.41**/**0.7709**	30.60/0.9141	**27.33**/**0.8318**
RBPN [9]	23.99/0.807	27.73/0.803	26.22/0.757	30.70/**0.909**	27.12/0.818
TDAN [44]	22.98/0.756	26.99/0.757	25.51/0.717	29.50/0.890	26.24/0.780
SAA (Ours)	23.81/0.8005	27.59/0.7962	26.15/0.7530	30.63/0.9077	27.05/0.8144
SAA+ (Ours)	23.97/0.8055	27.75/0.8031	26.25/0.7564	**30.81**/**0.9099**	27.20/0.8187

**Table 3:** Quantitative results of PSNR(dB)/SSIM of our proposed SAA networks compared with Bicubic and other state-of-the-art methods on **SPMCS**. The results on Y channel of YCbCr space are reported. The two best-performing methods are marked in **bold** and underlined respectively.

Clips	Bicubic	DBPN [27]	DRDVSR [2]	DUF [33]	SAA (Ours)	SAA+ (Ours)
car05_001	27.62	29.58	**32.07**	30.77	31.56	31.70
hdclub_003_001	19.38	20.22	21.03	**22.07**	21.88	22.03
hitachi_isee5_001	19.59	23.47	23.83	25.73	25.70	**26.21**
hk004_001	28.46	31.59	32.14	32.96	33.15	**33.32**
HKVTG_004	27.37	28.67	28.71	29.15	29.48	**29.55**
jvc_009_001	25.31	27.89	28.15	29.26	30.03	**30.34**
NYVTG_006	28.46	30.13	31.46	32.29	33.02	**33.31**
PRVTG_012	25.54	26.36	26.95	27.47	27.57	**27.67**
RMVTG_011	24.00	25.77	26.49	**27.63**	27.51	27.64
veni3_011	29.32	34.54	34.66	34.51	36.32	**36.82**
veni5_015	27.30	30.89	31.51	31.75	32.80	**33.10**
Average	25.67/0.726	28.10/0.820	28.82/0.841	29.42/0.867	29.91/.8708	**30.15**/**.8744**

**Table 4:** Ablation studies on the influences of each component of our proposed SAA. All models in this table are under configuration of F=7, M=5, C=6 and R=1.

Methods	$S_{a}$	$S_{b}$	$S_{c}$	$S_{d}$
MFE-CF?	×	✓	×	✓
$C^{3}AM$?	×	×	✓	✓
PSNR (dB)/SSIM (Y)	35.08/0.9192	35.31/0.9246	35.37/0.9251	35.44/0.9264
PSNR (dB)/SSIM (RGB)	33.35/0.9040	33.54/0.9093	33.57/0.9095	33.66/0.9113

**Table 5:** Ablation studies on the influences of SSR of our proposed SAA. All models in this table are with *F* = 7, *M* = 5 and *C* = 16.

Methods	$S_{e}$	$S_{f}$	$S_{g}$	$S_{d}$
Skips between SAAMs?	×	✓	×	✓
SSR inside each SAAM?	×	×	✓	✓
PSNR (dB)/SSIM (Y)	35.20/0.9238	35.42/0.9261	35.42/0.9262	35.44/0.9264
PSNR (dB)/SSIM (RGB)	33.26/0.9064	33.57/0.9103	33.64/0.9110	33.66/0.9113

**Table 6:** Influence of number of SAAMs (*M*) on our proposed SAA. All models in this table are with F=7, C=16 and R=1.

*M*	2	4	6	8	10
PSNR (dB)/SSIM (Y)	35.05/0.9213	35.29/0.9244	35.46/0.9265	35.60/0.9282	35.61/0.9284
PSNR (dB)/SSIM (RGB)	33.29/0.9056	33.52/0.9091	33.67/0.9114	33.81/0.9132	33.84/0.9137

**Table 7:** Influence of number of input frames (*F*) on our proposed SAA. All models in this table are with *M* = 5, *C* = 16 and R=1.

*F*	1	3	5	7
PSNR (dB)/SSIM (Y)	34.31/0.9099	34.69/0.9151	35.17/0.9225	35.44/0.9264
PSNR (dB)/SSIM (RGB)	32.60/0.8933	32.97/0.8993	33.40/0.9071	33.66/0.9113

**Table 8:** Influence of channels (*C*) on our proposed SAA. All models in this table are with *F* = 7 and *M* = 2.

*C*	16	32	64	128
PSNR (dB)/SSIM (Y)	35.05/0.9213	35.53/0.9273	35.95/0.9323	36.20/0.9351
PSNR (dB)/SSIM (RGB)	33.29/0.9056	33.76/0.9125	34.18/0.9183	34.42/0.9216

**Table 9:** Influence of numbers of residual blocks in each LRU (*R*) on our proposed SAA. All models in this table are with *F* = 7, *M* = 5 and *C* = 16.

*R*	1	2	3	4	5
PSNR (dB)/SSIM (Y)	35.44/0.9264	35.60/0.9282	35.82/0.9309	35.88/0.9314	35.91/0.9318
PSNR (dB)/SSIM (RGB)	33.66/0.9113	33.81/0.9133	34.01/0.9161	34.07/0.9168	34.10/0.9173

## Data Availability

Not applicable.
